# Identifying Strategies to Address Systemic Barriers to Blood Donation for South Asian Communities in Ontario: A Community-Based Approach

**DOI:** 10.3390/healthcare14111462

**Published:** 2026-05-26

**Authors:** Kelly Holloway, Poojan Joshi, Shruti Chandrashekhar Nadkarni, Aditi Khandelwal, Jasbir Singh, Maninder Dhaliwal, Lilet Raffinan

**Affiliations:** 1 Institute of Health Policy, Management and Evaluation, University of Toronto, Toronto, ON M5T 3M6, Canada; 2 Canadian Blood Services, Toronto, ON M5G 2M1, Canada; 3 Sant Nirankari Mission, Brampton, ON, Canada; 4 Blood Donation by Sikh Nation, Brampton, ON, Canada

**Keywords:** blood donation, South Asian, systemic barriers, qualitative, community-based

## Abstract

**Background/Objectives**: Building a donor base that reflects the diversity of Canada is essential to ensuring everyone has timely and reliable access to high-quality blood products. This qualitative research project aimed to both determine barriers to donation for diverse South Asian communities and seek feedback and guidance on proposed interventions to address those barriers. **Methods**: This study was guided by the principles of community-based participatory research and data was gathered and analyzed using constructivist grounded theory. We conducted eight in-person focus groups and four interviews. **Results**: Our findings indicate that barriers to donation are systemic. Barriers include inaccessibility, deferrals and negative donation experiences, lack of awareness and newcomer settlement challenges, social exclusion, navigating an unfamiliar donation system, and issues with access to appropriate care in health systems more generally. Participants proposed addressing these barriers through changes in the blood service, such as more convenient access to donation and improved cultural sensitivity and cultural comfort in donation centres, and also through changes in health systems more generally. Recommendations included sustained collaboration with communities to inform policies and practices based on cultural and social contexts. **Conclusions**: Our study of systemic barriers to blood donation for South Asian communities in Ontario indicates that barriers to donation are systemic. Participants proposed changes to blood services that would address some of these barriers. Where systemic barriers are attached to broader social structures, the strategies to address barriers will require longer-term considerations and resources.

## 1. Introduction

Blood services are responsible for collecting, testing and manufacturing blood and blood products, and for ensuring patients’ timely access to high quality products to support improved patient health outcomes and well-being. Currently, 74% of blood donors in Canada are white, resulting in a donor base that does not reflect the diversity of the Canadian population [1]. For some people with complex and ongoing red blood cell transfusion needs, specially selected red blood cell units are needed. These uncommon blood types are more likely to be found in donors of a similar ethnic background or ancestry. Additionally, to have a sufficient donor base for the future, the donor base must reflect the increasingly diverse population of Canada. Thus, building a donor base that reflects the diversity of Canada is essential to ensuring everyone has timely and reliable access to high-quality blood, platelet and stem cells, plasma, and organs and tissues. This research is a step towards building a blood service focused on equitable donation experiences and practices for an increasingly diverse Canadian population, focusing on South Asian communities. People of South Asian ancestry represent the largest racialized community in Canada, (2.6 million people; 7.1%) [2], and 5.85% of blood donors in Canada [3]. While slightly under-represented in blood donation, this group has a higher representation than other racialized communities and thus presents an instructive learning opportunity.

This qualitative research project aimed to both determine barriers to donation for diverse South Asian communities and seek feedback and guidance on proposed interventions to address systemic barriers to donation.

We build on existing empirical work examining systemic barriers to donation for under-represented racialized communities [4,5,6]. This work highlights the role of the blood service as an important intermediary between the donor and the recipient; one that can decide who is eligible to donate, the ways that donation is promoted, and the way in which the donation experience is structured [6]. There is limited research on South Asians living outside of South Asia related to blood donation [7,8]. Our own initial work on this topic indicated that barriers included accessibility, deferral criteria, gaps in awareness and knowledge about donation, language barriers, fears related to donation, and an absence of a blood operator in their communities [9]. We also learned that engaging South Asian communities requires ongoing communication, collaboration and trust-building with community leaders, including collaboration in creating strategies for recruitment, education and promotion of donation. The limitation of this previous work was a small sample size, comprising mostly men with a mean age of 53 years, and whose country of origin was India. The current manuscript represents our efforts to expand this study by undertaking more extensive engagement.

We draw on sociological literature about diverse South Asian communities in Canada to ensure that our study is informed and sensitive to the intersectionality of identities for these communities. For instance, we use the term “South Asian” with an understanding that it is a nuanced, contested, and an umbrella term which encompasses internal diversity and inequities [10,11]. Das Gupta’s work [12] emphasizes the intersectionality of transnational identities which include colonial, racialized, and gendered identities in the context of a neo-liberal globalized capitalist world economy. Thus, she integrates an intersectional approach to identity within a broader study of the political economy of global migration and transnationalism. This means understanding how identities are experienced differently in relation to features of the neo-liberal economy, such as precarious work, and globalized and domestic labour. Further, global political and racial economies produce inequalities that affect South Asian migrants, in both racialized and gendered ways [11].

This theoretical framework may seem tangential to blood donation, but as we discovered in our earlier work on this topic [9], this context speaks to what the communities identify as important; precarious employment, immigration status and racism were recognized as the most pressing concerns. These concerns and priorities are borne out in more recent literature on the South Asian diaspora in Canada [11,13,14]. This context can be what drives communities to seek a sense of belonging by contributing to what is perceived to be a national good, like blood donation. At the same time, that context can also be the reason why communities do not have the resources or the energy to seek accommodation or battle systemic barriers in yet another setting. This study expands and deepens our understanding of these barriers by working with communities, expanding the population of study, and choosing methodological tools that build trust and comfort for these communities.

## 2. Methodology

### 2.1. Community-Based Participatory Research

This study was guided by the principles of community-based participatory research (CBPR), which recognizes and aims to address the systemic and social disparities faced by specific communities [15]. This framework centers the active involvement of community members, not only as participants but also as guides and contributors to the research [16].

CBPR approaches have been regarded when working with historically disadvantaged communities such as immigrant or ethnic minority communities, who were the focus of this study [17,18]. In line with this approach, we partnered with three Canadian Blood Services community partners with significant South Asian representation. This included two longstanding partners, the Sant Nirankari Mission and Blood Donation by Sikh Nation, both of whom have led blood and plasma donation initiatives in partnership with CBS within their respective communities for a considerable length of time. We also collaborated with a more recent community partner, the Canadian Council of Muslim Women. These community partners were engaged in our project through a Community Advisory Team, which met 4–5 times throughout the year to advise researchers on how to conduct the study in a way that is sensitive to the needs of their community. They also guided recruitment efforts by promoting the call for participants and offered insights into how to communicate study findings. The community partners involved were offered an honorarium for their group.

To further include perspectives of younger South Asians, we connected with student groups representing South Asian communities across different universities and colleges in Southern Ontario. Ultimately, we partnered with a Pakistani student group, a Tamil student group, a South Asian women’s group and a South Asian research community group within three universities in Southern Ontario.

In keeping with a CBPR approach, we actively engaged community partners and student groups to plan for and conduct in-person focus groups in community and campus settings. Focus groups were an appropriate method for this study because they offered participants an opportunity to exchange ideas, express opinions and assert differences and commonalities [19]. Further, they are a way to reach under-represented communities and to talk about culturally sensitive matters, so members of a cultural group can explore a topic collectively rather than in a one-on-one encounter with a researcher, which may feel intimidating [20].

The study was approved by the Research Ethics Board at Canadian Blood Services (CBSREB# 2024.055) on 28 August 2024 and the University of Toronto (# 46923) on 5 September 2024.

### 2.2. Constructivist Grounded Theory

We use a qualitative methodology called grounded theory to concentrate on a behavioural process and advance an understanding of that process while staying “grounded” in the data. The purpose is to generate an explanation of a process, action or interaction shaped by the views of participants. We take a social constructivist approach to grounded theory using the work of Kathy Charmaz, which acknowledges that researchers and participants co-construct data [21].

### 2.3. Recruitment and Data Collection

We contacted and partnered with student groups between July and October 2024. This was followed by participant recruitment and data collection between October 2024 and February 2025 with support from community partners and student groups. The partners reached out to their community members using recruitment emails and posters provided by the research team. The posters included a link and QR code to a pre-screening questionnaire, assessing eligibility and availability for the study. Inclusion criteria involved interested individuals who self-identified as South Asian, and were 18 years or older in age, comfortable communicating in English and based in Ontario. We aimed to hold in-person focus groups within community settings to create a comfortable environment for participants. A suitable time, date and location for the in-person focus groups was determined in consultation with all eligible participants, after which the focus groups were conducted. In cases where some eligible participants were unavailable for focus groups, individual interviews were conducted to accommodate them. In other instances, focus groups were adapted into interviews when only one participant showed up. We also offered women-only focus groups, given that the literature and our earlier study found women sometimes wanted to discuss sensitive topics without the presence of men. Participants who took part in a focus group or interview were offered a $20 gift card. All in-person participants were offered a meal, and food was selected by community partners to accommodate each community’s cultural preferences and restrictions.

All participants gave their verbal consent for participation. Each focus group began with introductions and an icebreaker followed by questions about participants’ views on donation, cultural, religious and familial perspectives and influences on donation, systemic barriers to donations and participants’ feedback on proposed recommendations and strategies to address barriers within communities. Focus group participants were encouraged to respectfully interact and build on each other’s ideas and perspectives. This allowed us to identify shared or varying perspectives and experiences of donation and diverse barriers within communities. Some focus groups included exclusively long-term donors while others included individuals with little to no donation experience in Canada, allowing for varied perspectives and experiences with the blood system. These group compositions were not intentional but rather reflected our recruitment approach, which involved working alongside long-standing community partners (including long-term and past donors) and university campus student clubs (including donors and those with little or no donation experience).

The focus groups were conducted by three moderators. The questions were divided into sections and each moderator focused on specific sections in all focus groups. Interviews were one-on-one, and each member of the research team conducted interviews. In seven focus groups, the Principle Investigator KH acted as lead moderator. She is a white woman and works as a social scientist at CBS. KH focused on questions around participants’ experiences donating with CBS and suitable ways in which the blood operator can engage with participants’ communities. PJ (Moderator 2), a Research Assistant and first-generation Indian woman, led focus group discussions related to barriers to donation. SN (Moderator 3), a Research Assistant and first-generation Indian immigrant, focused on questions around familial, religious and cultural perspectives participants held about donation. One focus group was moderated by PJ and SN.

As one moderator focused on leading the discussions, the others took detailed notes. These notes included information about the general topics the participants discussed, focus group dynamics, and any verbal and non-verbal details the researchers noticed during, prior or after the focus groups. The researchers reflected on these notes during data analysis. Upon completion of focus groups and interviews, audio recordings were transcribed verbatim by a professional transcriptionist. De-identified (to remove identifying information) transcripts were uploaded to NVivo 14 for qualitative analysis.

### 2.4. Data Coding and Analysis

Our study was guided by constructivist grounded theory [22]. During data collection we iteratively read transcripts and data collection notes from the focus groups and interviews to familiarize ourselves with the data and continually revise the interview and focus group guides. Then, using NVivo 14, SN coded the transcripts, staying close to the data using descriptive codes, and regularly consulting with KH and PJ about codes and patterns generated from the data [21]. This process is referred to as the “constant comparative method” which involves continual comparison of data with data, code with code, to find similarities and differences, grouping codes into categories [22]. Alongside coding, we wrote reflexive memos detailing our insights, and notable patterns identified during the coding process. The research team met weekly during data collection and analysis to discuss emerging concepts, relationships between and among the data, and insights related to the literature and theories informing this study. This process of moving between the data and theory is called abduction [22]. Throughout this process, codes were refined into categories and ultimately into broad analytical themes. We use Hennink and colleagues’ (2017) concept of “meaning saturation” to determine the point when the researchers fully understand the issues and where no further dimensions, nuances or insights are being discovered [23]. We reached a point in our data collection and analysis where we felt we understood the meanings conveyed by participants, with adequate complexity given the diversity of the sample. In line with our participatory approach, we presented these themes to our community partners who offered feedback to help refine the themes and ensure the findings reflected their communities’ perspectives. While our analysis focuses on these themes, we have also reflected on our experiences conducting focus groups in Supplementary File S1.

## 3. Results

We conducted eight in-person focus groups, four in-person interviews, and 2 interviews via Microsoft Teams for those unable to attend in person. Two focus groups were organized for women only. We collected participant demographic information including age, gender, immigration status, country of origin, and donation status (see Table 1). First-generation was defined as immigrants to Canada; second-generation, defined as children of immigrants to Canada; and third-generation, defined as grandchildren of immigrants to Canada. In total, 34 participants took part in the study, ranging in age from 18 to 73 years. Eighteen participants identified as women and 16 as men. Most participants were first-generation immigrants to Canada (n = 25) and most had not donated or tried to donate blood (n = 19). In our analysis when we refer to participants with “donation experience” we mean those who have donated or tried to donate whole blood or plasma in Canada within the preceding 12 months.

Our results indicate that barriers to donation are systemic. Participants described barriers that were attached to practices of the blood operator, such as language barriers and location and hours of operation of donor centres. They also described barriers that were connected to systems such as immigration, education and healthcare in Canada. Participants’ recommendations to address barriers to donation include sustained collaboration with communities to inform policies and practices based on cultural and social contexts. Where systemic barriers are attached to broader social structures, the strategies to address barriers will require longer-term considerations and resources.

Below, we discuss barriers to blood donation among participants with donation experience in Canada, participants with little or no donation experience in Canada, and health-related barriers discussed among both groups.

### 3.1. Barriers Among Participants with Donation Experience

#### 3.1.1. Inaccessibility

Participants who had donated blood identified inaccessibility and negative donation experiences as major barriers to donation, especially for community members not comfortable communicating in English. Specific challenges included understanding eligibility criteria, medical terminology in screening procedures, the donation process, and communicating questions and concerns during donation appointments. Furthermore, community partners expressed frustration that, despite repeated advocacy for interpreters and translated materials, there had been no significant changes to reduce this barrier. Inaccessibility also extended to the hours of operation and location of donation centres, which participants described were far from where they lived and worked. Long travel times and scheduling challenges such as having to take time off from work and/or school further contributed to this inaccessibility.

#### 3.1.2. Deferrals and Negative Donation Experiences

Several participants also identified donor deferrals and negative staff interactions during donation events as barriers. Specifically, communication related to deferrals was a major concern. Participants noted a lack of support and guidance during health-related deferrals for whole blood donation (e.g., iron deficiencies, travel to malaria-endemic regions, and/or medication use) and some described missing or inconsistent information from staff about eligibility and return timeline for donation. These experiences led some participants to feel dismissed and undervalued. Although these participants mentioned that this would not impact their own willingness to donate, they could see how new, less experienced, or non-English speaking donors may be deterred from returning to donate.

While these participants highlighted how ongoing barriers shape their experiences of donation, insights from those with little or no previous donation experience revealed additional and sometimes overlooked barriers to donation.

### 3.2. Barriers Among Participants with Little or No Donation Experience

#### 3.2.1. Lack of Awareness & Newcomer Settlement Challenges

Lack of awareness was a significant barrier among participants with little or no donation experience in Canada. These participants noted an absence of discussion of donation within their families, communities, and social circles. They also reported that promotional materials were not visible in their everyday environments, and when present, information about eligibility, the donation process, and the need and impact of blood donations were insufficient. All participants also noted a general lack of awareness and understanding of other forms of donation such as plasma, stem cell, and platelet donation in their communities.

Notably, many participants attributed their limited awareness of blood donation to their families’ settlement experiences in Canada, during which other priorities were more pressing. Participants with first-generation immigrant parents recounted how their parents’ early lives in Canada were spent striving to meet immediate needs and family responsibilities. As one younger participant explained,

“…my parents at least, I think with most, like immigrants…would come here with not much money… so they would have their mind on, you know, taking care of their kids or finding work, and then they wouldn’t find chances to donate blood. And maybe, and that like leads to, like me and my siblings like, never getting taught about it.”

[FG1P2, second generation immigrant, no donation experience in Canada]

Another participant who is now a donor helped offer more insight by connecting this lack of awareness to broader struggles of settlement and adjustment for members of their community,

“… our community, the minute they come to Canada, the struggle starts, you know, […] my parents had to do nightshifts, my dad has a PhD, my mom was a teacher back home, he wasn’t able to get a job here, so he had to do factory work, security jobs, I wouldn’t see him for a week, cause he was working two jobs at a time. So this was not a priority at all at that time. Times were tough. But I think as […] things got a little bit better, you just kind of learn as you go and you realize what’s important; if we were still in that state, I don’t think I would be here, talking to you about this. I think we would still be struggling.”

[FG4P1, first-generation immigrant, active donor]

These participant reflections demonstrate how the demands of financial and social stability take precedence for newcomers, and the awareness and capacity to donate increase as these struggles subside.

Challenges associated with newcomer settlement were further echoed by international students who described their own struggles of balancing multiple priorities such as school, jobs, and navigating changing and unfamiliar immigration and health systems in Canada. As one participant reflected,

“I was like OK, so why haven’t I donated? And when I think about it, I’m like, you know like, I came to Canada and I was…[in an] impoverished situation, honestly, like from all sides, not just financially but, like, it was really hard, it was cold, it was a very new environment, and I didn’t understand norms, rules, X, Y, Z. So, and it was hard, like PhD was hard, I didn’t have any time…”

[FG9P2, international student, no donation experience in Canada]

#### 3.2.2. Social Exclusion

Several participants highlighted feelings of social exclusion in Canada. For some, it was tied to their temporary residence status and feelings of not belonging. However, one participant who had lived in Canada for decades also echoed this sentiment of not belonging. This was further complicated by growing anti-immigrant sentiments, experienced through everyday occurrences of racism, rise in hate crimes, and policy such as reducing the number of immigrants admitted to Canada [24,25]. For international students and other newcomers, feelings of exclusion and disconnection were tied to their temporary resident status and general challenges within Canada. These experiences weakened participant’ willingness to engage in donation behaviours. One participant noted while recalling her experience as an international student,

“…there has been a lack of even acceptance, and there has a been a point where, maybe, it’s almost seen like, this is not our country, then why do it [donate blood]? You know. Like because if we are not accepted, if we are looked at in such a bad light, then why do it? That, and there’s also a barrier to, I’d say, medical assistance […] where, even if you need a blood test, you’ve gotta wait two weeks, you know. So again, it connects with the point where we said, more information, where, I don’t know, if I go in to give my blood, do I have to wait three hours? And what happens afterward?”

[FG8P2, international student, no donation experience in Canada]

These experiences demonstrate how social and systemic barriers directly affected participants’ willingness to engage in initiatives like donation.

#### 3.2.3. Navigating an Unfamiliar Donation System

In addition to feelings of social exclusion, participants with little or no donation experience also discussed navigating an unfamiliar donation system as a barrier. Participants compared their experiences with family-replacement systems in their countries of origin to the voluntary nonremunerated donation (VNRD) system in Canada. In countries such as India and Bangladesh, participants described donating blood in response to direct requests from family, friends, and social networks, (family replacement donation) which made donation feel more urgent. In contrast, participants noted that they were often unfamiliar with the VNRD system in Canada, and this unfamiliarity sometimes contributed to perceptions that there was no urgent need for blood. When combined with other barriers, such as limited awareness of the need for blood and a lack of targeted outreach, this unfamiliarity reduced motivations to donate. Some participants also noted that the absence of direct requests to donate, and insufficient information about who needs blood created a sense of distance from the donation system, leading them to perceive donation as primarily for those already embedded within health and social systems in Canada.

Thus, barriers for participants with little or no prior donation experience extend beyond a general lack of awareness. They reflect broader challenges of migration and settlement, social inclusion, and unfamiliarity with health and blood systems that shape how newcomers perceive and experience donation. For many, donation can feel unfamiliar, inaccessible, and rooted in systems they are still learning to navigate.

### 3.3. Health Systems as a Barrier

Discussions of health barriers were raised primarily among women, who mentioned several health issues that impacted their ability to donate blood, such as anemia. Participants spoke of these issues as being common in their communities but still remain stigmatized and dismissed, both within families and in clinical settings. They also shared that they received little or no guidance when seeking help to improve their iron levels and were often dismissed or offered ineffective or inaccessible treatments. This included their family physicians, who did not offer sufficient information or culturally appropriate and accessible treatment for low iron. In the context of blood donation, this also included donor centre staff, as one participant noted asking for an informational pamphlet several times, and left feeling dismissed after they did not receive one, even when this resource was available. Given these challenges, one participant indicated that they relied on health professionals in India for more culturally appropriate, affordable, and accessible care, including access to dietitians and iron infusions.

Unrelated to donation, participants described Canada’s healthcare systems as difficult to navigate and unresponsive to the ongoing needs of their community. They noted struggles to receive adequate care for health issues such as diabetes, high blood pressure, and low iron. This included long wait times for appointments and diagnostic testing, long wait times in Emergency departments, and slow or inadequate follow up from physicians to address their issues. For example, one participant recalled her visit to the Emergency room:

“I had the same experience, like I had to go into the Emergency Room, I was having a severe asthma attack, I was sitting there coughing for almost four hours, like well into the morning, and you know, eventually I had my inhalers with me, I kept taking my medication, and eventually, how long did I cough for? I could have had a heart attack, right?...after four hours, something, I started feeling a little bit better, and I was able to breathe, I walked out of there and as I was walking out, I asked them, what do you charge for this? Mind you, I have no insurance, because I’m not a student anymore… so, I asked them, and they said that, no you won’t be charged because you didn’t see a doctor, great, next week, I get a letter with an invoice, [laughing], $1100.00 for sitting there, and almost having, you know, a heart attack, from a severe asthma attack, I was not looked at…”

[FG8P3, international student, no donation experience in Canada].

This participant’s experience shows how newcomers, including students, may perceive and experience healthcare systems as inaccessible and slow, where their health concerns could go unaddressed, but also where the burden of this care is on them, both in terms of managing their health concerns and in taking on unexpected financial costs. Another participant also described waiting months to see a specialist only to leave without effective solutions, leading them to rely on family and social networks or medical care abroad.

These barriers were further impacted by a broader mistrust of Western medicine and medical systems, rooted in personal and intergenerational experiences. A few participants spoke about how negative medical experiences shaped their skepticism toward Western healthcare. Some participants were more comfortable with traditional or home-based remedies over Western medicine. Participants talked about a preference to treat minor illnesses with natural remedies rather than pharmaceuticals, which reflected both cultural and intergenerational traditions as well as a mistrust in formal medical systems.

These systemic barriers demonstrate that participants did not see the blood service as completely separate from the broader healthcare systems, and so their reservations and concerns about these systems affected how they viewed donation. One participant explained, when healthcare feels inaccessible to them, it is difficult to feel motivated to contribute to it through blood donation. Lastly, these questions also extended to concerns about their health post donation, particularly for international students who may lack health coverage or find themselves in precarious positions between study permits and permanent residency.

### 3.4. Addressing Systemic Barriers

Our study found that blood services can address systemic barriers based on knowledge of cultural contexts and collaboration with diverse communities. Recognizing the diverse barriers that South Asian communities experience in donation, we asked participants what improvements would support them and their communities in a blood donation context. This analysis is not divided between donors and non-donors because participants’ suggestions were consistent across both groups.

### 3.5. Changes in the Blood Service

#### 3.5.1. Convenient Access to Donation

Participants recommended actionable strategies to improve accessibility to blood donation. These recommendations included policy changes to allow language support and interpreters during donation appointments, offering mobile donation events within community settings to support a comfortable, familiar, and convenient donation experience, and sufficient, updated, and widely disseminated information regarding eligibility, upcoming donation events in community and campus settings, and opportunities to follow up post donation to counter concerns related to health and potential adverse effects.

Participants indicated that making donation accessible is the responsibility of the blood service, and not the individual donor. They emphasized the need to minimize the burden of donation by removing any logistical barriers such as travelling to donor centres. As noted in the discussion on barriers, donating blood often required participants to actively manage multiple priorities and make specific arrangements to be able to donate. Given this barrier, many participants indicated that donation would be more accessible if opportunities were integrated into their existing routines. One specific recommendation was to offer mobile donation events, when possible, particularly in spaces where community members already congregate, such as schools, workplaces, and religious/spiritual spaces. This would facilitate donation for those who have busy lives but are motivated and willing to donate. Furthermore, community members with long-standing relationships with the blood operator also reported a need for additional donor centers in cities with larger South Asian populations. These high-footfall spaces are also beneficial for new immigrants who are not yet familiar with public transportation systems.

In one focus group, participants expressed that community members may be more receptive and motivated to donate if they learn about donation experiences from other community members. Further, providing advance notice of upcoming mobile donation events and eligibility criteria would minimize unnecessary travel efforts and deferrals.

Participants emphasized that clearly defined eligibility and deferral criteria are essential. This information is particularly critical for recent immigrants, deferrals in Canada may be distinct from those in their country of origin. Those who did have experience with donation reported that the current resources have ambiguous wording. For example, “travelling to malaria-endemic regions” was seen as ambiguous. While blood services often also offer a list of the countries, participants did not find this information accessible. Additionally, participants noted that most of their communities travelled to South Asia often to meet family or for events, in such cases, they are frequently deferred due to recent travel. Therefore, donor participants in the study asked for clear communication about this deferral, and to consider reviewing and changing it.

Lastly, participants recommended offering opportunities for donors to follow up with staff post donation and post deferral (e.g., a post-donation helpline). The ability to access support from knowledgeable staff was important for people with health concerns or temporary residents without sufficient healthcare coverage.

#### 3.5.2. Improved Cultural Sensitivity in Donation Centres

Participants highlighted a need for cultural sensitivity and kindness from staff present at donor centers. Negative experiences and unpleasant interactions during donation would deter donors from returning, especially for people who do not speak English.

“you know, extended family, neighbours, some of them are not well-versed in English, and there’s no way they can fill out that questionnaire by themselves. They have to have a family member or support to explain things to them […] I think cultural sensitivity is required. Every culture is different. Being in the Brown community, you know, it’s, it’s tough, it’s difficult, and it can get quite chaotic, in that volunteer space, when I was there, I saw how busy it got. So I think a little bit of cultural sensitivity, would be required so that they stay patient, with the donors. And, like, [FG4P2] said that they don’t realize that them being impatient could trigger someone in such a negative way, these things, we’re all, we run our lives by emotions, right? Everything around us revolves around emotions, so this is so important to make sure that the community feels cared for, and they’re encouraged to donate blood, and they don’t have any, too negative experiences”

[FG4P1]

This participant pointed to several challenges in staff interactions at mobile donation events. She discussed the unique needs of community members who may face language barriers. Further, when mobile donation events take place in the community this can pose challenges for staff who are adjusting to that space—a situation that could require increased patience and understanding.

The patience and kindness also need to be enough for the community to feel welcome in donation spaces. For another participant, a lack of such a space led to a decreased motivation to donate. The participant wanted to be recognized and valued for her voluntary contribution, but her negative experience led to a feeling of being under-appreciated, ultimately souring her desire to continue donating. Beyond a welcoming environment, verbal and non-verbal appreciation from staff are also necessary for donors in these spaces.

Sensitivity from staff is particularly important during deferrals. Many donors take significant pre-donation efforts, for example, travelling long distances, taking unpaid time off work, arranging childcare, or increasing iron-rich foods prior to their appointments to be eligible to donate. Given these efforts, how the staff approaches the deferral is important. One participant said she were treated differently when her hemoglobin was tested:

“… I wouldn’t say that it was, they were rude to me, but it was just different, like they were disappointed. And it wasn’t the first time it happened. I wasn’t given any resources to look at, how can I improve my iron, what can I do? […] So then I tried to eat like iron-rich foods and whatnot, so then in two months I went again to donate regular blood and I think they treated me worse than how I was treated at the plasma centre, cause they saw that I had a probation, and then I came back, and my iron was still low.”

[FG4P1]

The treatment this participant received for having low hemoglobin made her feel like she disappointed the staff because of her ineligibility. Instead of being offered resources, the participant was left feeling frustrated and without the proper resources to address their reason for deferral. As such, participants recommended that staff communicate about deferrals in a private space, and in a non-judgmental and constructive way. Even when deferred, the participants wanted to feel needed as donors. One participant recommended creating an encouraging response to deferrals by reframing them as temporary hurdles and then supporting them in crossing such hurdles.

#### 3.5.3. Cultural Comfort

Participants with and without donation experience suggested that the blood service could support a more culturally comfortable sensitive donation experience for South Asian communities. This included sustained community outreach, co-creating promotional materials, and in-centre/mobile donation infrastructures that are considerate of the cultural nuances of diverse south Asian communities.

A central recommendation in creating cultural comfort was the presence of the blood service within the South Asian community. Creating strong and long-term partnerships with diverse South Asian community organizations, being present for important community and cultural events, social media engagement with South Asian groups and increased presence in cities with a higher South Asian population was commonly recommended.

Participants also advocated for more mobile donation opportunities in the community where they could be comfortable and support each other. They recommended places of worship such as temples, gurudwaras, churches and mosques for community-led awareness and outreach efforts. This would require attention to the religious norms and rules of religious spaces (e.g., separate sections for men and women at mobile donation events). Given there may be additional explicit and implicit rules, the following participant recommended working alongside champions within the religious organizations to coordinate donation events.

“… we must make our temples, our gurdwaras, our places of worship, into health hubs, you know, and that’s not happened. If we did that, because you know, they have so much infrastructure, like they have huge spaces, huge halls, you know, you can give a small fee for you know, appreciation, if needed, but they have the infrastructure, they have, you know, people who take care of the facilities, things like that, so I would say, why not leverage on that, because that’s where the community is, instead of kind of building new communities and drives, go where they are, you know, so then half your work is done. You’re not paying a lot for resources, you’re not paying a lot for people to get the buy-in, you know, things like that, they’re already there.”

[FG8I5, first-generation immigrant, past donor].

Some Muslim participants also recommended finding Imams (religious leaders) who can be such champions because some community members may consult a religious leader about donation practices. Participants also indicated a need for more South Asian representatives from the blood service, both in person and in promotional materials such as social media campaigns.

“Just to add to what FG4P1 said, I think when you see person with the same culture, same background, automatically you become more comfortable.”

[FG4P2, first-generation immigrant, active donor].

Specifically, these promotional materials can be tailored to highlight the importance and need for different forms of donation within diverse South Asian communities. Therefore, creating promotional materials with South Asian representation and in South Asian languages would be beneficial and impactful.

While many participants highlighted the need for representative staff who could speak their language and tailored outreach, they recognized that it was not always possible given the diversity within South Asian communities. To address this, they recommended call helplines, and potential use of technology for quick and low-cost translations of resources. Markedly, the participants expressed the size and complexity of the pre-donation questionnaire as a barrier; therefore, a reduced size of the questionnaire and increased simplicity in the questionnaire’s wording was suggested. In English and translated materials participants wished to see more colloquial language in addition to medical terminology which may be challenging for new and less experienced donors.

Lastly, participants recommended exploring new avenues for outreach for different demographics within their communities. For example, participants noted middle-aged and older adults in their communities relied on WhatsApp as their primary mode of communication and information sharing whereas younger community members relied on Instagram and TikTok. Aligning outreach efforts and mobile donation events with cultural and religious events was also identified as a critical factor. For example, participants noted that approaching a mosque during Ramadan would not be effective as the community would be fasting and unable to donate. Alternatively, reaching out before Ramadan, or around celebratory times like Diwali or Nagar Kirtan would be more appropriate as the community had already gathered and may be looking for ways to give back to their community.

Ultimately, the participants emphasized that for outreach and awareness to be effective, it must be appropriate, familiar, and respect the community’s cultural practices.

### 3.6. Changes in the Health System

Participants spoke candidly of their trust in South Asian doctors and nurses who have gone out of their way to support community members. Individuals may also be more comfortable asking questions to medical professionals than CBS representatives. Involving South Asian medical professionals also reduces the language barrier that identified by several participants. The sense of familiarity with the language extends to the sense of familiarity with the individual speaking the language and helps create trust with the individual,

“Like, if, this is a cultural thing too, like in terms of like, if you go see a doctor, like you know how they would say, hi, how are you? Like, in Tamil though, it’s kind of friendly in the sense that, the way, just like, the way you act and the way you talk to someone else, is kind of different than, how you would say it in English, right”.

[FG3P1, second generation immigrant, no donation experience in Canada].

South Asian medical professionals were also recommended for outreach efforts.

“So I think, if we have, if, and we do have a lot of, I’d say, Brown doctors and everything, in the system, but, it’s just, maybe finding the right person, if I find a right doctor here, and if that doctor encourages me and says, you know what, if, whenever you’re feeling well, I think you’re at a good stage, health-wise, you’re looking OK, you should go and donate. And if I trust that doctor, I will a 100 percent go and donate, it wouldn’t, it would negate all of those factors, it would negate all of the barriers that we spoke about, all of the racism, discrimination, that would have, that would take a back seat, because somebody who I trust, is asking me to do something.”

[FG8P2]

Across focus groups, particularly women discussed low iron in relation to donation. The blood service tests for hemoglobin at every donation, and if it is too low the donor is deferred until they meet the threshold for donation. One of the more common reasons for lower hemoglobin is low iron, but there may be other causes of low hemoglobin. This was an important issue for participants because iron-deficiency is a common issue for South Asian women. Participants noted that the current institutional guidance on iron health (unrelated to donation) often fails to resonate with many within the community following a vegan or vegetarian diet. Further, participants did not always have positive experiences with iron supplementation. For blood donation, participants advocated for promotional and education material that accommodates the cultural specificities of the community,

“Say for example, you have a lot of people who have low iron, what if you guys, as, this is just purely promotional material, talk about, OK, say you’re South Asian, what can you eat from your culturally based diet, that would improve that, and that would help you donate. What signs do you have to show that you have good blood? Something relating to our culture, to the aspect of donating itself.”

[FG1P5, first-generation immigrant, no experience with donation in Canada].

Having resources that consider the daily diets of South Asian communities, which may differ from other Canadians, would support the community in addressing health-related reasons for deferrals.

## 4. Discussion

This examination of systemic barriers to blood donation for South Asian communities suggests that barriers exist both within blood services’ operational policies (for instance, language and accessibility) and also are attached to the broader social world (for instance, systems of immigration and health care). We build on our earlier findings [9] which demonstrated that for South Asian communities in Canada, barriers include lack of accessibility, language, deferral criteria and lack of awareness. At the same time, we expand and deepen these findings by working closely with diverse communities to hold in-person focus groups and expanding our recruitment efforts to include more women and youth.

Describing barriers to blood donation as systemic means not only pointing to particular policies of the blood operator, but also demonstrating how barriers are integrated into the way in which social systems operate for racialized communities in Canada. Our analysis highlights how donation is inaccessible specifically for immigrant communities who are struggling to make a life in Canada, where policies of the blood operator that are designed for all donors made this population feel dismissed and undervalued because of language or lack of information that was culturally appropriate. Broader barriers for those who had little or no donation experience were even more intimately tied to their identities as newcomers and visible minorities in Canada. Our analysis leverages scholarly work on the political economy of global migration and transnationalism [12,26] so that we can point to how these barriers do not only exist at an individual level but are woven through the experience of being South Asian in Canada, with the challenges that come with finding meaningful employment free of discrimination, supporting education for younger generations, and navigating complex health systems that can further marginalize these communities, sometimes in ways that are gendered.

This study’s findings offer evidence that cultural context has an impact on interest, awareness, and experiences of donation, particularly as recent immigrants face uncertainty about their status in Canada, which makes it difficult to experience belonging. This echoes literature on social inclusion and citizenship in a blood donation context where the willingness to donate blood is linked to feelings of social inclusion and recognition within the host society [27]. When donation systems reinforce these barriers, donation is perceived as something that is inaccessible. Participants in this study described how that accessibility was particularly relevant for immigrant populations, because adjusting to Canada involves layered struggles with work, education, language and discrimination [28,29]. For this reason, donation is not a priority, especially if it is inconvenient.

Importantly, this experience is not static. Participants engaged in discussions about how their community had created opportunities for donation that was culturally relevant, or how they learned about blood donation through the education system in Canada. Younger participants sought out opportunities to better understand donation in a Canadian context and then discussed donation with elders in their community to understand if it is compatible with their faith. Thus, perhaps the most crucial finding of this study is that it is possible for these communities to see donation as accessible, meaningful and valuable for them, even in a Canadian context. They spoke to the specific changes that would facilitate donation for their communities.

Some of our findings have direct implications for the blood service. To counter the barriers related to settlement challenges and lack of understanding and awareness about donation, participants recommended strategies to improve accessibility. They advocated for more donation opportunities in their community, more detailed and accessible information about how to donate and the blood services to support a culturally inclusive environment, increased language support and community-based outreach efforts. Culturally inclusive policies may both enhance access and help diverse communities feel a greater sense of belonging in the donation space, as environments that recognize and celebrate diversity have been shown to enhance immigrants’ sense of inclusion [30]. Such an inclusive space might be particularly important today given changes to national immigration policy and the rise in anti-immigrant sentiments [24].

In response to negative experiences while donating, particularly related to deferral, participants wanted staff to be sensitive to their cultural context and to their experiences as South Asians in Canada. This requirement could also be connected to broader systems of discrimination and feelings of social exclusion experienced by people who are racialized.

Our findings also indicate that when blood donation is not seen as separate from broader healthcare systems, it reduces trust, motivation and willingness to donate for those who have experienced these systems as inaccessible, ineffective, and dismissive. Our study supports existing evidence of barriers to health care for immigrants to Canada, including communication, cultural, knowledge-related, socioeconomic, health care system and individual-level barriers [31]. While some barriers, such as hospital wait times, are shared with the broader Canadian population, navigating this complex system without the same fluency in English or cultural customs is a particularly salient challenge for immigrants (ibid). Given these structural and systemic barriers for South Asian communities in Canada’s health systems, studies have emphasized the need for cultural competence and humility from healthcare providers [32]. In many ways, our study resonates with these findings in the broader literature on immigrant communities and access to health care.

Some participant recommendations about health can be taken up by blood services. For instance, more culturally specific information about the relationship between hemoglobin and low iron, clear communication about donation eligibility for people with common health conditions and medications, and outreach and follow up that acknowledges the needs of South Asian communities could help to reduce barriers and increase trust. Other recommendations are beyond the scope of the blood operator. Questions about access to health care and cultural competency of care providers in health systems was particularly relevant for women participants. Their concerns reflect the challenges they face in accessing adequate and culturally appropriate care in other areas [33,34,35]. Similarly, for international students especially women faced challenges navigating health systems, staying healthy, limited on-campus availabilities and difficulty travelling to see a doctor.

Finally, our study builds on scholarly work about the South Asian diaspora in Canada which documents discrimination and racism towards these communities throughout Canada’s history and also recently [11,36]. This study took place during increasing anti-immigrant sentiments in the US and Canada. Participants in this study situated their experiences within this context, as well as broader social and political systems, and described the ways in which discrimination and exclusion have affected their understanding and orientation towards blood donation. That context can mean that not all immigrants see donation as a priority because they are focused on survival, and that experiences of donation can be seen as unwelcoming if not sensitive to cultural norms. This coupled with the understanding that donating could lead to weakness, or even potentially illness, makes it into a risk that does not make sense given other challenges they are facing. Parents warned their children of this risk. And international students thought about that risk when they reflected on their access to health care in Canada.

### Strengths and Limitations

Our study demonstrates rigor by presenting rich descriptions and explanations offering a complex picture of this phenomenon [37]. Our sincerity and reflexivity about positionality are outlined carefully in our reflection presented in Supplementary File S1. The credibility of our study [37] is strengthened by our collaborative approach with community partners, who offered feedback about the data from their lived experience.

This is a qualitative study of systemic barriers to blood donation for South Asian communities in Southern Ontario, and therefore the findings are specific to that context. While this study offers in-depth knowledge about this topic, a limitation of this work is that it is not generalizable beyond this context. Further studies could explore this population in other Canadian contexts, or beyond Canada. Another limitation of the study is that while we were interested in studying this population’s experience with other forms of donation such as plasma and stem cell donation, the participants had very little experience and knowledge of those forms of donation, and thus our findings were limited to blood donation. Furthermore, we did not purposefully collect data on participants’ religious, spiritual, or faith-based affiliations. Instead, we encouraged participants to self-define the communities with which they identified. While participants offered recommendations on how best to engage with their religious, spiritual, or faith communities, including appropriate approaches and timing, a more in-depth examination of these dimensions remains an area for future research.

Finally, eligibility criteria included being comfortable participating in English. This criterion was established due to practical constraints in our capacity to conduct and translate focus groups and interviews in multiple languages. As a result, this study does not reflect the perspectives of South Asian individuals who are not comfortable communicating in English. This limitation further reinforces the language barrier identified in this study. We acknowledge this important gap and hope to secure funding to offer opportunities to participate in this research in multiple languages in the future.

## 5. Conclusions

This study of systemic barriers to blood donation for South Asian communities in Ontario indicates that barriers to donation are systemic. Participants proposed changes to blood services that would address some of these barriers. While blood services do have some of the resources that participants identified, and while they attempt to communicate and mitigate any potential risk, the perception among participants that the support was not always apparent or available is noteworthy. Acknowledging the complexity of these experiences and concerns can be an opportunity for blood services to re-examine existing materials on donor safety, eligibility, and deferral, and more generally, to acknowledge that barriers to donation are systemic for these communities, thus they must be addressed systemically. The participants in this study were clear that the work of the blood service begins with more active engagement with communities—to learn about their experiences, the barriers from their perspectives, and to orient donation to under-represented racialized communities by promoting accessibility and cultural comfort. Where systemic barriers are attached to broader social structures, the strategies to address barriers will require longer-term considerations and resources which fundamentally re-imagine how health systems serve diverse communities. Where efforts in blood systems are successful, they could serve as a model for health systems more generally.

## Figures and Tables

**Table 1:** Participant Demographics.

**Gender**	
Women	18
Men	16
**Age**	
Age range	18–70+
Mean age	39
Majority age range	18–44
**Migration Status**	
First generation	25
Second generation	6
Third generation	1
Unknown	2
**Country of origin**	
India	22
Pakistan	6
Sri Lanka	2
Bangladeshi and Pakistani	1
Bangladesh	1
Other (South Asian, Arab)	2
**Donation Status**	
Donated/Tried to donate	15
Have not donated/have not tried to donate	19

## Data Availability

The data that support the findings of this study are available on request from the corresponding author. The data are not publicly available due to privacy or ethical restrictions.

## Supplementary Materials

The following supporting information can be downloaded at: https://www.mdpi.com/article/10.3390/healthcare14111462/s1, File S1: Reflections on advantages and challenges of using focus groups to engage South Asian communities.
