# Supplementary material for: Identifying Strategies to Address Systemic Barriers to Blood Donation for South Asian Communities in Ontario: A Community-Based Approach
Source: Healthcare (Basel). 2026 May 26;14(11):1462. doi: 10.3390/healthcare14111462 (PMC13256442; doi:10.3390/healthcare14111462)
Supplement: Supplementary file 1 [file healthcare-14-01462-s001.zip › healthcare-4211545-supplementary.pdf]

Supplement S1: Reflections on advantages and challenges of using focus groups to engage South Asian communities:

This supplementary document highlights the researchers' reflections on advantages and challenges of conducting focus groups in the community with members of the South Asian community in Canada. These insights are shaped by our positionalities – two of us are South Asian immigrant women in Canada,[1] one of us is a white woman, and two of us work for one of Canada's blood services.

Advantages:

- Community partners placed cultural and social significance in community gatherings for donation. Focus groups in that community setting recognized the importance of meeting in a place that was familiar and safe. These spaces were effective in fostering open dialogue on donation related issues.
- Consulting community partners about culturally appropriate food offerings allowed participants to experience the familiarity of gathering within their communities.
- Meeting in person in a safe space appeared to help participants voice negative experiences they had encountered as South Asian individuals within Canada's donation and healthcare systems. For example, in one focus group, a new international student's feelings of not belonging were echoed by a long-term immigrant. Through their shared experiences they were able to have a conversation about donation that extended into reflections of their identities, belonging, and communities.
- In focus groups that involved participants who belonged to similar religious, cultural, and linguistic groups, participants were able to rely on each other for language support, to collaboratively clarify religious nuances, and draw from shared cultural knowledge. Across both community and student groups, participants frequently paused to confirm the appropriate translation of specific terms with one another. Given that language remains a persistent barrier within the South Asian community, particularly among older members, the presence of other community members who shared linguistic and cultural

[1] This supplementary document has primarily been led by XX and XX.

backgrounds helped foster more inclusive and comfortable participation, even when English was the primary language of communication.

- The moderators who also identified as South Asian shared some similar experiences. This sense of shared understanding may have created a space where participants felt more comfortable discussing sensitive or personal issues.

Challenges:

- While focus groups can offer opportunities for participants to connect with each other, and validate and build on each other's perspectives and experiences, these interactions can also be shaped by specific group contexts, such as familiarity and comfort with the participants in the focus group, and social standing and influence within the group. This was especially relevant in focus groups where participants already knew one another and were connected through the same community group/organization. In these settings, some participants may feel comfortable enough to speak openly whereas others may not, especially when some members are older, more established, or hold more senior roles within the organization. As a result, what participants choose to share (or not share) may be shaped by their own social standing within the group.
- Community organizations can play an important role for newcomers as they become a source of practical support such as offering advice and connections for employment and housing, as well as social comfort, acceptance, and a sense of belonging. This means that newer members may feel a greater need to align with the perspectives and experiences of the organization/community. As such, they may not feel entirely comfortable expressing disagreement or "negative" experiences of donation, especially if they differ from more established members. To address this challenge, future research can offer focus groups within similar levels of seniority/social standing, and/or offer confidential follow-up options for newer members.
- There were some logistical challenges in organizing in-person focus groups. Given that many community connections and communications occur digitally, participants who identify as belonging to community groups and organizations are not necessarily bound by their physical location. At the same time, defining "community" is complex, particularly within diverse South Asian communities, where shared identity is shaped by

region, country, religion, language, and migration history. Together, these factors made it difficult to coordinate a time and location that was accessible for all eligible and interested participants and, in some cases, limiting who was able to attend. This required additional planning and flexibility, such as offering virtual interviews for participants unable to attend in-person focus groups. Furthermore, this has implications for those who were able to reach, who were able to participate, and how community is operationalized in community-based participatory research.

- While we have highlighted how language barriers were reduced in the focus-group setting, we also observed the benefits of allowing participants to speak in their first languages. In one particular focus group, participants who had only recently met began conversing in a shared native language, which the moderators also spoke. The conversations during this time fostered much comfort and connection more quickly.
